# A Pilot Study of Dietetic, Phenotypic, and Genotypic Features Influencing Hypertensive Disorders of Pregnancy in Women with Pregestational Diabetes Mellitus

**DOI:** 10.3390/life13051104

**Published:** 2023-04-28

**Authors:** Karina dos Santos, Eliane Lopes Rosado, Ana Carolina Proença da Fonseca, Gabriella Pinto Belfort, Letícia Barbosa Gabriel da Silva, Marcelo Ribeiro-Alves, Verônica Marques Zembrzuski, Mario Campos, Lenita Zajdenverg, Michele Drehmer, J. Alfredo Martínez, Cláudia Saunders

**Affiliations:** 1 Programa de Pós-Graduação em Nutrição, Instituto de Nutrição Josué de Castro, Universidade Federal do Rio de Janeiro, Avenida Carlos Chagas Filho, 373-Bloco J 2° Andar, Cidade Universitária, Rio de Janeiro 21941-902, Brazil; 2 Escola de Nutrição, Universidade Federal do Estado do Rio de Janeiro, Avenida Pasteur, 296, Prédio 2, 3° Andar, Rio de Janeiro 22290-240, Brazil; 3 Laboratório de Genética Humana, Instituto Oswaldo Cruz, Fundação Oswaldo Cruz, Pavilhão Leônidas Deane, Avenida Brasil 4365, Rio de Janeiro 21040-360, Brazil; 4 Instituto Nacional de Infectologia Evandro Chagas, Fundação Oswaldo Cruz, Avenida Brasil 4365, Rio de Janeiro 21040-360, Brazil; 5 Departamento de Clínica Médica, Faculdade de Medicina, Universidade Federal do Rio de Janeiro, Avenida Carlos Chagas Filho, 373-Bloco K, 2° Andar, Cidade Universitária, Rio de Janeiro 21941-902, Brazil; 6 Programa de Pós-Graduação em Epidemiologia e Programa de Pós-Graduação em Alimentação, Nutrição e Saúde, Faculdade de Medicina, Universidade Federal do Rio Grande do Sul, Rua Ramiro Barcelos 2400, Porto Alegre 90035-003, Brazil; 7 Precision Nutrition and Cardiometabolic Health Program, IMDEA Food Institute (Instituto Madrileño de Estudos Avanzados en Alimentación), Crta. de Canto Blanco, n 8, E-28049 Madrid, Spain

**Keywords:** pregnancy in diabetes, pregnancy-induced hypertension, preeclampsia, DASH diet, nutrigenetics

## Abstract

Hypertensive disorders of pregnancy (HDP) are a leading cause of maternal and perinatal morbimortality. Dietetic, phenotypic, and genotypic factors influencing HDP were analyzed during a nutrigenetic trial in Rio de Janeiro, Brazil (2016–2020). Pregnant women with pregestational diabetes mellitus (n = 70) were randomly assigned to a traditional or DASH diet group. Systolic blood pressure (SBP) and diastolic blood pressure (DBP) were measured during prenatal visits and HDP were diagnosed using international criteria. Phenotypic data were obtained from medical records and personal interviews. Genotyping for FTO and ADRB2 polymorphisms used RT-PCR. Linear mixed-effect models and time-to-event analyses were performed. The variables with significant effect on the risk for progression to HDP were: black skin color (adjusted hazard ratio [aHR] 8.63, *p* = 0.01), preeclampsia in previous pregnancy (aHR 11.66, *p* < 0.01), SBP ≥ 114 mmHg in the third trimester (aHR 5.56, *p* 0.04), DBP ≥ 70 mmHg in the first trimester (aHR 70.15, *p* = 0.03), mean blood pressure > 100 mmHg (aHR 18.42, *p* = 0.03), and HbA1c ≥ 6.41% in the third trimester (aHR 4.76, *p* = 0.03). Dietetic and genotypic features had no significant effect on the outcome, although there was limited statistical power to test both.

## 1. Introduction

Hypertensive disorders of pregnancy (HDP) are a major cause of maternal and perinatal mortality and morbidity worldwide, mostly occurring in under-resourced settings. Monitoring blood pressure is key for screening for HDP during prenatal care, helping to protect maternal and infant health [1].

Women with pregestational diabetes mellitus (DM) are at high risk of HDP. About 7% of all pregnancies are affected by preeclampsia [2], but in women with DM its incidence can reach 20% [3]. Other recognized high-risk factors for HDP are chronic hypertension, multifetal gestation, nulliparity, preeclampsia in previous pregnancy, renal disease, and autoimmune disease. Additional moderate-risk factors are obesity, family history of preeclampsia, unfavorable sociodemographic characteristics, and age 35 years or older [4].

Having a healthy diet is believed to decrease the risk of preeclampsia by 33% [5] and to improve perinatal outcomes [6,7,8]. The DASH (Dietary Approach to Stop Hypertension) diet was designed to prevent and treat hypertension, which can be explained by its composition rich in calcium, magnesium, potassium, mono- and polyunsaturated fatty acids, dietary fibers, and antioxidants [9]. The adherence of pregnant women with cardiometabolic disorders to the DASH diet can reduce the risk of obstetric and perinatal complications, including the incidence of preeclampsia [10], although this effect was not yet investigated in women with pregestational DM.

As a multifactorial disease, there is also a strong genetic component in hypertension during pregnancy, and several genes have been linked with HDP [11]. Genes that interact with diet are of particular interest to nutrigenetic investigations that aim to provide personalized nutrition for individuals at higher risk for the disease [12]. The adrenoceptor beta 2 gene (ADRB2) encodes the beta-2-adrenergic receptor, which responds to sympathetic stimulation for vasodilation and nitric oxide release in the endothelium, thus affecting blood pressure control [13,14]. In the United States, one month of intervention with the DASH diet had a different effect on blood pressure reduction in adults with stage 1 hypertension, according to the ADRB2 genotypes for two common polymorphisms, rs1042713 (G/A) and rs1042714 (C/G), indicating that the benefits of the diet were influenced by genetic characteristics [15].

Polymorphisms in the fat mass and obesity-associated (FTO) gene are strongly associated with obesity, type 2 DM, and elevated blood pressure [16]. The FTO polymorphisms rs9939609 (T/A) and rs17817449 (T/G) have been associated with obesity in the Brazilian population [17]. The FTO genetic predisposition to hypertension seems to be associated with risk of preeclampsia in European and Central Asian women [18]. However, the negative effects of the FTO genotype on the metabolic phenotype can be modified by gene-environment interaction and counteracted by a healthy diet [19].

The precise influence of diet, phenotype, genotype, and their interactions on the development of HDP remains unclear. Accordingly, the aim of this study was to investigate the dietetic, phenotypic, and genotypic features that influence the development of HDP in women with pregestational DM using two different diets: DASH diet or a traditional Brazilian diet.

## 2. Materials and Methods

### 2.1. Subjects

A nutrigenetic trial was conducted as part of the randomized controlled clinical trial named DASDIA (DASh diet for pregnant women with DIAbetes), at the Maternity School of the Federal University of Rio de Janeiro, Brazil (2016–2020, Brazilian Clinical Trials Registry RBR-4tbgv6). 

The eligibility criteria were: women with pregestational type 1 or type 2 DM, diagnosed before or during pregnancy [20], aged >18 years at conception, in less than the 28th week of pregnancy, single fetus, no use of alcohol, tobacco, or drugs, no sexually transmitted diseases (e.g., HPV, genital herpes, syphilis), no psychiatric diseases (e.g., depression, anxiety, eating disorders), and no complications related to DM (e.g., diabetic retinopathy or nephropathy). Women who had treated and controlled hypothyroidism (TSH 0.1–2.5 mUI/L in the first trimester or 0.3–3.0 mUI/L in the second trimester, using levothyroxine) or chronic hypertension (diagnosed before pregnancy or until 20 weeks’ gestation, using methyldopa) were eligible. The participants were randomly assigned to one of the two parallel study groups—DASH diet or traditional diet, and blinded to the dietary allocation. Randomization was performed using a list of random numbers generated by a computer software (Microsoft Excel 2016).

According to the institution’s protocol, all the participants were treated with insulin therapy as prescribed by a physician for their individual needs. Women with gestational DM were not included. The women who initiated their prenatal care before the 16th week of pregnancy were prescribed a daily dose of aspirin (100 mg) as a preventive measure against preeclampsia [21].

### 2.2. Diet Groups and Nutritional Guidance

Individual nutritional guidance was offered for all participants, from the date of inclusion in the study until the last prenatal appointment. Duration of intervention was calculated as the time between inclusion in the study and delivery.

DASH and traditional diets had the same macronutrient content (45–55% carbohydrates, 15–20% protein, and 25–30% total fat), the same sodium content (2400 mg), but differences in the content of fatty acids, fiber, calcium, magnesium, and potassium (Table 1). The traditional diet was the same diet that was routinely prescribed for pregnant women with DM receiving prenatal care at the study site. The DASH diet prescribed a higher quantity of whole grains, fruits, vegetables, and low-fat dairy products. It also included a daily serving of nuts. The original version of the DASH diet was translated and adapted for the DASDIA trial to consider the characteristics of Brazilian pregnant women with DM, as detailed elsewhere [22,23,24].

Daily energy intake was calculated for each participant, considering their age, physical activity, pre-pregnancy body mass index (BMI), and expected gestational weight gain. A meal plan, including a list of food equivalents, was provided and explained in detail by the registered dietitian to all the study participants.

In order to improve dietary adherence, the women allocated in the DASH diet group received a bottle of extra virgin olive oil (500 mL) on their first visit, and a can of powdered skimmed milk (280 g) and a pack of nuts (150 g) and seeds (200 g) at each visit. The participants of the traditional diet group received a bottle of extra virgin olive oil (500 mL) on their first visit, and a can of powdered semi-skimmed milk (300 mg) and a packet of oats (250 g) at each visit.

To assess dietary adherence, 24 h dietary recalls were used, as well as a tool with four evaluation items: (1) quantity of food consumed (portions); (2) food groups consumed (variety); (3) meals consumed (number and time of day); and (4) gestational weight gain (adequate when no more than 20% more or less than recommended). The score could vary from 0 to 4 points [25] and was stratified into low-to-moderate adherence (<2 points) and high adherence (≥2 points). The adherence score used for the present analysis was the one measured at the visit closest to delivery and thereby reflecting the longest possible time of exposure to the intervention.

When calcium intake was estimated to be <900 mg/day, a supplement with 500 mg calcium carbonate was prescribed as a preventive measure against preeclampsia after the 20th week of pregnancy, independent of the group [26]. 

### 2.3. Blood Pressure Measurements and Outcome Assessment

Blood pressure was measured at each prenatal visit by a trained assistant nurse using an aneroid sphygmomanometer, with the woman seated after a minimum 15 min of rest. SBP and DBP were registered in the medical records, accessible to the whole health care team, and this information was used for the study. Mean blood pressure (MBP) was calculated by: MBP = [SBP + (2 × DBP)]/3. 

The main study outcome was the development of HDP. The institution’s protocol for HDP diagnosis follows international guidelines from the American College of Obstetricians and Gynecologists (ACOG) [4]. Gestational hypertension was defined as new onset of SBP ≥ 140 mmHg or DBP ≥ 90 mmHg at or after 20 weeks gestation. Gestational hypertension accompanied by proteinuria (≥300 mg in a 24-h urine sample) or new-onset organic dysfunctions was diagnosed as preeclampsia. When women who had chronic hypertension developed proteinuria, they were classified as having superimposed preeclampsia.

As HDP diagnosis is a medical assignment, it was assessed by consulting the medical records and confirming with the attending physician. Time until gestational hypertension (GH) or preeclampsia was estimated by linear interpolation between different measurements. The last registered gestational blood pressure measurement was taken upon admission for delivery.

### 2.4. Genotyping

The genotypic characteristics investigated were the common variants of FTO (rs9939609 T/A and rs17817449 T/G) and ADRB2 (rs1042713 G/A and rs1042714 C/G). The participants provided a saliva sample, and genomic DNA was isolated from buccal epithelial cells [27]. Polymorphisms were genotyped by RT-PCR using TaqMan^®^ assays (ThermoFisher, Carlsbad, CA, USA). Reactions were performed in 10 μL volumes, containing DNA (2 μL), Universal Master Mix (5 μL), TaqMan Genotyping Assay specific for each polymorphism (0.25 μL), and MiliQ (2.75 μL). Amplification was carried out in a StepOne^®^ Plus Real Time PCR System (ThermoFisher) using the number of cycles and temperatures recommended by the manufacturer. Positive and negative controls were included on the plates.

### 2.5. Covariates

The covariates were: age (years), DM type (1 or 2), education level (9 years of schooling, 12 years of schooling, higher education), marital status (married/single), employment status (employed/unemployed), per capita income (total household income divided by the number of residents, in US dollars), housing conditions (adequate (regular garbage collection, piped water, and sewage collection) vs. inadequate (absence of one or more of above)), pre-existing chronic disease (hypothyroidism, yes/no; chronic hypertension: SBP ≥ 140 mmHg and/or DBP ≥ 90 mmHg, diagnosed before pregnancy or until 20 weeks gestation, yes/no), history of previous HDP (none/preeclampsia diagnosed in a previous pregnancy of the same woman), serum glycated hemoglobin concentrations (HbA1c), and parity (number of previous births). The data were retrieved from the medical records and gaps were filled during prenatal visits using a structured questionnaire. The participants self-reported their skin color (white/brown/black/yellow) and years since onset of DM. Gestational age was obtained by ultrasound.

Pre-pregnancy BMI was calculated as self-reported pre-pregnancy weight (kg) divided by the square of their height measured at first prenatal visit (m^2^), and classified as underweight (BMI < 18.5 kg/m^2^), normal weight (BMI 18.5–24.9 kg/m^2^), overweight (BMI 25.0–29.9 kg/m^2^), or obesity (BMI ≥ 30.0 kg/m^2^). Gestational weight gain was calculated as weight at admission for delivery minus pre-pregnancy weight.

The cutoffs for SBP, DBP, and MBP were >130 mmHg, >80 mmHg, and >100 mmHg, respectively, based on the American College of Cardiology/American Heart Association (ACC/AHA) criteria for stage 1 hypertension [28]. The numerical variables age, gestational weight gain, SBP, DBP, and HbA1c were categorized by the median of the overall sample to compare the risk for HDP between the higher values (above the median) and the lower values (below the median). 

### 2.6. Statistical Analyses

The Mann–Whitney U test was used to compare continuous numerical variables, and Fisher’s exact test was used for categorical demographic and baseline clinical variables. Genotype and allele frequencies of each variant were determined by direct counting, and the Chi-squared test was used to evaluate deviation from Hardy–Weinberg equilibrium. Paired linkage–disequilibrium patterns were determined for each gene using R squared statistics (r^2^ cutoff ≥ 0.8).

Linear mixed-effect models were used to analyze SBP, DBP, and MBP longitudinally against the diet (traditional or DASH), the genotypes or minor allele frequency (MAF), and their first-order interactions. Longitudinal patterns were modeled by directly specifying a natural spline term (with two knots) in the models. To take account of the lack of independence of the longitudinal measurements for each woman, this term was also included as a random effect. The main effect was also corrected for confounding variables. The results were presented graphically and in tables. 

The gestation period was divided into four: <6.5 weeks, 6.5–19.5 weeks, 19.5–32.5 weeks, and >32.5 weeks. Marginal mean values and their 95% confidence intervals (CI) were estimated. All the other variables in the multiple linear models were maintained in their mean values or equal proportions at conception, week 13 (end of the first trimester), week 27 (end of the second trimester), and at the day of delivery. Contrasts were constructed from these estimated mean marginal effects. *p*-values were corrected by the number of pairwise comparisons by the Holm–Sidak method. Statistical significance was set at *p* < 0.05.

Genotypes were compared using additive, dominant, and recessive models. Haplotype frequencies, or rather allelic phases, were estimated by expectation maximization (EM algorithm), and estimation uncertainty was included in the statistical models used for the time-to-event analyses in the form of weights. The haplotype analyses used the most common haplotype in our population as the reference.

The incidences of GH or preeclampsia were analyzed on the basis of the follow-up time, calculated as the duration from the most likely date of conception to the most likely date of the outcome. Incidences and their 95% CI were estimated according to asymptotic standard errors calculated from a Gamma distribution. The women who did not experience the outcome were considered in the analyses from the most likely date of conception until the date of delivery. The results of the time-to-event analyses were presented in the form of hazard ratios (HR) and 95% CI, and the risks of progression to the events described above were estimated via Cox proportional hazard models. The assumption of risk proportionality was tested using correlation analyses and Chi-squared tests based on Schoenfeld’s scaled residuals and transformed survival times.

In the time-to-event analyses, as in the longitudinal analyses, the effects of the genetic characteristics of interest were corrected for phenotypic characteristics with at least one suggested association (*p*-value ≤ 0.1) with the outcome of interest, and the marginal effects were presented in the form of adjusted hazard ratio (aHR). Each polymorphism was evaluated in the additive, dominant, and recessive models, but some polymorphisms had null incidence of HDP for the genotypes analyzed in the additive or recessive models, so the results were presented only in the dominant model (comparison between MAF carriers vs. MAF non-carriers).

Statistical analyses were performed using R (version 4.1.1) and its “lme4”, “splines”, “genetics”, and “survival” packages. The power analysis and sample size estimates were performed post hoc using an R code (available at: http://powerandsamplesize.com/Calculators/Test-Time-To-Event-Data/Cox-PH-2-Sided-Equality, accessed on 1 December 2021). Given that the overall prevalence of the event was 23% (16/69), the frequency of minor allele carriers was 0.35, the mean hazard ratio was 2, and alpha = 0.05, the minimum sample size for the Cox proportional models estimate of power (1—beta) of 0.8 was 312. Given our limited sample size (n = 70), the statistical power of the analysis was 26.16%, so we considered this a pilot study.

## 3. Results

**Figure 1:**
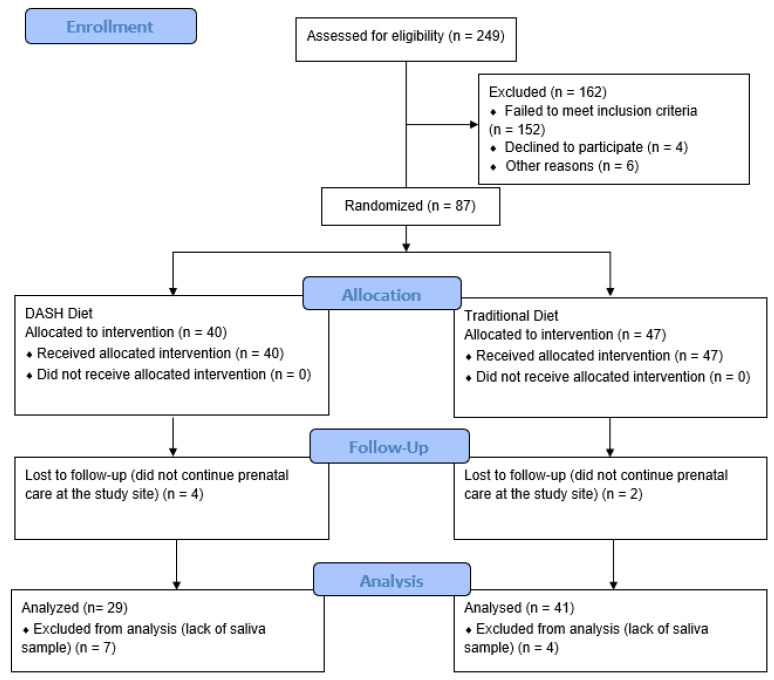
Flowchart of the study.

Eighty-seven women participated in the DASDIA clinical trial, 70 of whom had sufficient data for the analyses and were therefore included in the present study. Twenty-nine were allocated to the DASH diet group and 41 to the traditional diet group (Figure 1). 

The median age of the participants was 32 years (IQR 25.7–36.0) and median gestational age at randomization was 15 weeks (IQR 11.1–20.1); 51.4% of the cases of DM were type 1. Most of the women had pre-pregnancy excessive weight: 35.7% had overweight and 35.7% had obesity. Ten women (14.3%) had chronic hypertension, treated with methyldopa. Median SBP was 110 mmHg (IQR 100–120) and median DBP was 70 mmHg (IQR 62–80). The distribution of the variables was homogeneous among the two dietary groups (Table 2).

The median duration of intervention was 22.50 weeks (IQR 15.50–26.04). Most of the women attended the six scheduled appointments (n = 38, 54.3%) or at least five of them (n = 16, 22.9%). In the visit closest to delivery, dietary adherence was high for 39.5% of the participants on the traditional diet and 40.7% of the participants on the DASH diet.

The genotypic frequencies of the polymorphisms in the FTO and ADRB2 genes were similar between the groups (Table 3). 

**Figure 2:**
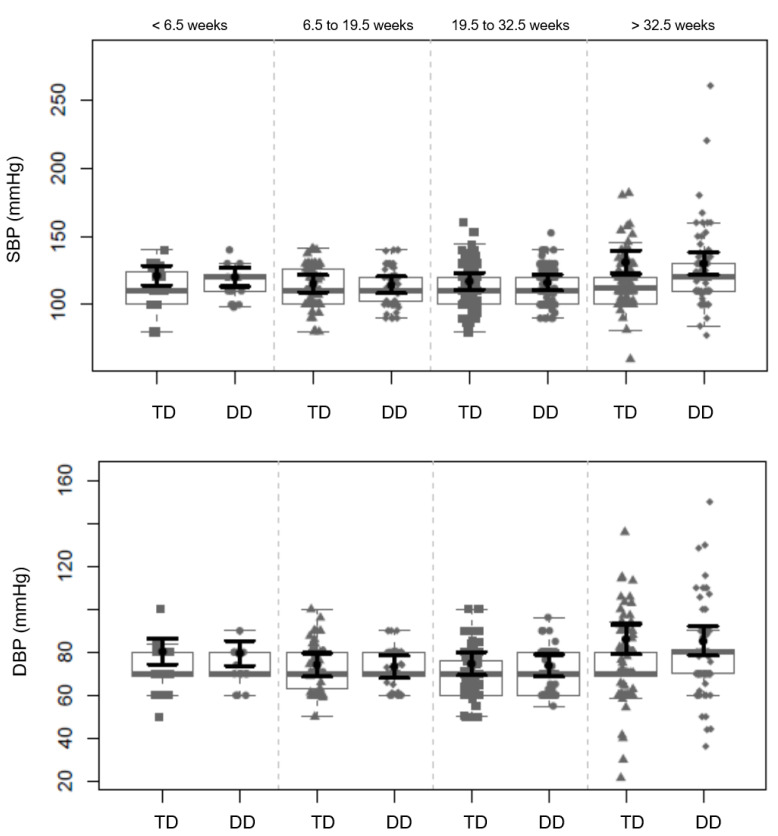
Trajectory of systolic blood pressure (SBP) and diastolic blood pressure (DBP) during pregnancy for the traditional diet (TD) and DASH diet (DD) groups. Linear mixed-effect models adjusted for type of diabetes, previous hypertensive disorders of pregnancy, pre-pregnancy body mass index, chronic disease (hypertension or hypothyroidism), and housing conditions.

There was no difference between the groups in terms of blood pressure trajectory during pregnancy (Figure 2, Supplementary Table S1), with SBP and DBP increasing after 32.5 weeks of pregnancy in both groups. Analyzing the effect of the genotypes on blood pressure during pregnancy, the carriers of the A allele of FTO rs9939609 had higher SBP, DBP, and MBP than the non-carriers in the overall sample and in the traditional diet group, but not in the DASH diet group, although this effect was borderline for statistical significance (Supplementary Table S2). No significant effect of FTO rs17817449 or ADRB2 rs1042713 and rs1042714 genotypes was found, nor any interaction of these genotypic characteristics with diet on blood pressure trajectory, but for rs17817449 and rs1042714, SBP, DBP, and MBP were higher in the MAF (G) allele carriers throughout pregnancy, compared to the non-carriers (Supplementary Tables S3–S5).

Diet was not found to have a significant effect on the development of GH or preeclampsia. Sixteen (22.86%) women developed HDP: nine (21.95%) from the traditional diet group and seven (24.14%) from the DASH diet group (*p* 0.87). Overall, there were five (7.14%) cases of GH and 11 (15.71%) of preeclampsia, with no statistical difference between the groups (Table 2). The cases of GH developed between 30 and 37 weeks of pregnancy. Seven cases of preeclampsia developed before 37 weeks of pregnancy, five of which were in the traditional diet group, and two cases of preeclampsia developed before 34 weeks (one in each diet group). Three of the ten women with chronic hypertension developed superimposed preeclampsia (30%), all in the traditional diet group. All cases of preeclampsia were diagnosed with proteinuria. There was a difference in the gestational age of childbirth between the women with HDP and the women without HDP: 36.9 weeks (34.8–38.0) and 38.0 weeks (37.3–38.1), respectively (*p* = 0.02).

Irrespective of dietary group, the phenotypic characteristics associated with progression to HDP (Table 4) were black skin color (aHR 8.63, 95% CI 1.85–40.18, *p* = 0.01), history of preeclampsia in previous pregnancy (aHR 11.66, 95% CI 2.22–61.07, *p* < 0.01), peak values of MBP > 100 mmHg during pregnancy (aHR 18.42, 95% CI 1.38–245.98, *p =* 0.03), SBP ≥ 114 mmHg in the third trimester (aHR 5.56, 95% CI 1.09–28.42, *p* 0.04), DBP ≥ 70 mmHg in the first trimester (aHR 70.15, 95% CI 1.43–3450.19, *p* = 0.03), and HbA1c ≥ 6.41% in the third trimester (aHR 4.76, 95% CI 1.14–19.86; *p* 0.03). All 16 women who developed HDP had at least one DBP measurement above 80 mmHg, and 12 (75%) had at least one SBP measurement above 130 mmHg during pregnancy.

Sociodemographic characteristics: marital status, education level, employment, housing conditions, and household income were not related to the development of HDP in our sample (*p* > 0.05). Thus, number of gestations, parity, years since diabetes diagnosis, and history of miscarriages were also not associated with the outcome (*p* > 0.05, data not shown). 

In the adjusted analysis, no association was found between the FTO and ADRB2 polymorphisms and progression to HDP (Table 5) in the haplotype analyses for either FTO rs9939609:rs17817449 (TT/AG/AT) or ADRB2 rs1042713:rs1042714 (AC/GC/GG) (Table 6).

## 4. Discussion

Among the dietetic, phenotypic, and genotypic characteristics assessed, only the phenotypic ones were associated with the development of HDP in our sample. The non-modifiable risk factors were black skin color and history of preeclampsia in previous pregnancy. The risk factors that were potentially modifiable during pregnancy were DBP ≥ 70 mmHg in the first trimester and HbA1c ≥ 6.41% in the third trimester. The peak values of MBP > 100 mmHg and SBP ≥ 114 mmHg in the third trimester were probably manifestations of disease.

Although a suggestive effect of the FTO rs9938609 A allele on the trajectory of blood pressure was found, this effect did not influence progression to HDP in the time-to-event analysis. Comparing the two types of healthy diets (traditional and DASH diet), no difference in the risk of developing HDP and no diet-gene interaction was found, but in the DASH diet group there were no cases of superimposed preeclampsia. 

Asemi et al. (2013) found a slight decrease in SBP (–2.6 mmHg) after four weeks of intervention with the DASH diet in 34 Iranian normotensive women with gestational DM [29]. Courtney et al. (2020) found that higher adherence to the DASH diet during pregnancy decreased DBP and MBP in the first and third trimesters in 511 healthy Irish women [30]. In our sample, there was no difference in the trajectory of blood pressure between the diet groups. The fact that the dietary interventions were not introduced early in pregnancy for all the participants may be one reason for their apparent lack of effect.

The blood pressure trajectory of our participants was similar to that observed in healthy pregnancies, with an increase in SBP and DBP towards the end of pregnancy [31], which may reflect benefits from the two types of diet. Nutritional guidance is a powerful non-pharmacological approach in the management of DM during pregnancy to avoid adverse outcomes [20]. Both the traditional and the DASH diet fostered healthy eating patterns based on guidance by registered dietitians, to meet the energy and macro and micronutrient requirements of each pregnant woman, so benefits could be expected from both of them.

Fulay et al. (2018) found no association between adherence to the DASH diet and incidence of HDP in a retrospective cohort of 1760 healthy pregnant women in the United States [32], but Jiang et al. (2019) found a lower frequency of preeclampsia in 85 Japanese pregnant women with chronic or gestational hypertension when initiating the DASH diet before 28 weeks of pregnancy, compared with a healthy control diet (43.2% vs. 65.9%, *p* 0.036) [33]. The incidence of preeclampsia in women with chronic hypertension was higher in Jiang’s study (30%) than in our sample. 

It is interesting to note that we had a higher percentage of women with chronic hypertension allocated in the DASH diet group (20.7%) than to the traditional diet group (9.7%), but none of them developed superimposed preeclampsia, while all three in the traditional diet group did. We also found a higher percentage of GH in the DASH diet group than in the traditional diet group (10.34% vs. 4.88%) and a lower percentage of preeclampsia in the DASH diet group than in the traditional diet group (13.79% vs. 17.07%). Although this result was not statistically significant, it suggests that the DASH diet may offer a protective effect against progression to preeclampsia for the subgroup of women with chronic hypertension or GH, but we are not able to confirm this hypothesis in the present study. 

In a sample of adults from the U.S. with stage 1 hypertension, the GG carriers of ADRB2 rs1042713 and rs1042714 were less responsive to the DASH diet in terms of blood pressure decrease after one month’s intervention [15], but this was not corroborated in our sample of pregnant women with DM from Brazil. Pregnancy is a state of intense adaptation in the cardiovascular system, including changes in the renin–angiotensin-related pathways [34], which could result in different effects of gene–diet interaction in the maternal metabolism.

FTO is the gene most related to obesity, and the adverse cardiometabolic effects of its polymorphisms are mostly mediated by excess adipose tissue [35]. We found a suggestive effect of the A allele of rs9939609 on blood pressure trajectory during pregnancy, which was higher in the traditional diet group but not in the DASH diet group. However, this A allele was not confirmed as a risk factor for progression to HDP in our sample. 

Being black-skinned is well-documented as a risk factor for hypertension, with black patients often having the lowest rates of BP control in clinical settings. Some studies have attributed this not only to ancestry, but also to socioeconomic inequalities [36]. We found the black-skinned participants were also at higher risk to develop HDP than the brown-skinned ones, irrespective of their socioeconomic indicators, suggesting that ancestry may be an important component. Regarding the evaluated polymorphisms in FTO and ADRB2 genes, there was no difference in MAF according to skin color.

Using clinical factors to identify women at high risk of preeclampsia early in pregnancy is valuable for mitigating adverse impacts on maternal and infant health. Bartsch et al. (2016) [37] evaluated more than 25 million pregnancies in a meta-analysis including 92 studies and found that a history of preeclampsia in a previous pregnancy was the clinical factor with greatest pooled relative risk of preeclampsia (relative risk 8.4; 95% CI 7.1–9.9). Our results corroborated these findings, but they did not corroborate other recognized risk factors, such as nulliparity, chronic hypertension, obesity, and maternal age [4]. However, we should recall that all the participants had pregestational DM, which is also a high-risk factor for preeclampsia.

Glycemic control before and during pregnancy is strongly recommended to prevent preeclampsia and other adverse outcomes, such as congenital abnormalities, macrosomia, and preterm birth in pregnancies with DM. Higher HbA1c in the third trimester (>6.41%) increased almost five-fold the risk of progression to HDP, but when it occurred in the first and the second trimesters, it had no impact on this risk.

For women with pregestational DM and chronic hypertension, the target BP is 110–135/85 mmHg to reduce the risk of HDP and minimize impaired fetal growth [20]. In our sample, the median BP values during pregnancy were mostly within this target, but DBP ≥ 70 mmHg in the first trimester—higher than the median of the overall sample—was a risk factor for HDP. 

Darwin et al. (2021) found the risk of preeclampsia was three times higher in women with ACC/AHA stage 1 hypertension (130 mmHg SBP and/or 80 mmHg DBP) in the first trimester compared with normotensive women [38]. Bello et al. (2021) found that the use of the ACC/AHA criteria to diagnose hypertension during pregnancy resulted in a 20.8% improvement in the appropriate identification of future preeclampsia [39], but McLaren et al. (2021) found that the rate of preeclampsia among women with hypertension by the ACC/AHA criteria was not significantly different from the rate among women with hypertension by the ACOG criteria [40]. 

In our sample, all the women who developed HDP had at least one DBP measurement above 80 mmHg, and 75% of them had at least one SBP measurement above 130 mmHg during pregnancy. Based on these figures, we hypothesize that in women with pregestational DM, the ACC/AHA cut-offs for BP may be used to warn of risk for HDP—something that deserves more investigation.

This study has three main limitations. The first is its small sample size and thus its limited statistical power. However, it offers the first results from a study applying a nutrigenetic approach to evaluating HDP as an outcome. We believe that knowledge on possible gene–diet interactions during pregnancy may benefit maternal and infant health with precision nutrition, especially in high-risk pregnancies, such those of women with pregestational DM. Bigger samples in future studies may help to clarify some hypotheses we presented here.

The second limitation is that the COVID-19 pandemic forced us to make adaptations to maintain the follow-up of the study. In 2020, prenatal visits to physicians were maintained at the study site, but for six women (8.6% of the sample) who were already enrolled in the study at the beginning of the pandemic lockdown period, nutritional guidance and data collection for the research were conducted by virtual means (telemedicine). The virtual visits used the same study protocol as the regular in-person visits. No new participants were admitted to the study once lockdown was in place.

Finally, it should be noted that preeclampsia is more hazardous to maternal and fetal health than GH [4]. Thus, early onset preeclampsia (before 34 weeks of pregnancy) may have more severe consequences than the disease developed after 34 weeks, with more adverse maternal and fetoplacental conditions and more severe complications [41]. We analyzed all cases of HDP together (GH and preeclampsia at any time of pregnancy) given our study design and sample, but these differences may be considered in designing future studies in this field, since dietetic, phenotypic, and genetic characteristics may interact differentially according to disease severity.

## 5. Conclusions

In our sample, black skin color, a history of preeclampsia in a previous pregnancy, DBP ≥ 70 mmHg in the first trimester, HbA1c ≥ 6.41% in the third trimester, and peak values of MBP > 100 mmHg during pregnancy were the phenotypic risk factors for HDP. No difference was found between the DASH diet and the traditional diet or between the genotypes in terms of risk for progression to HDP. However, our results need to be interpreted with caution because of their limited statistical power.

Identifying women at higher risk of HDP using clinical evaluation is a feasible strategy to improve prenatal care and reduce maternal and neonatal morbidity and mortality among women with pregestational diabetes. Future studies combining dietetic, phenotypic, and genotypic characteristics in larger samples should be implemented to clarify the multifactorial etiology of HDP, which may improve prenatal care and thus maternal and infant health.

## Figures and Tables

**Table 1:** Composition of the diets used in the study for an example of 2100 kcal daily energy intake.

	DASH Diet	Traditional Diet
Calcium	2280 mg	1500 mg
Magnesium	496 mg	315 mg
Potassium	4418 mg	4081 mg
Fiber	55 g	42 g
Saturated fatty acids	7.2% E *	9.7% E
Monounsaturated fatty acids	9.2% E	8.5% E
Polyunsaturated fatty acids	5.6% E	2.8% E

* % E = percentage of daily energy intake.

**Table 2:** General characteristics of the study participants (Rio de Janeiro, Brazil, 2016–2020).

	Overall n = 70	DASH Diet n = 29	Traditional Diet n = 41	*p*-Value *
Age (years)	32 (25.7–36.0)	34 (28–37)	31 (25.0–35.0)	0.28
Gestational age (weeks)	15.0 (11.1–20.1)	16.0 (10.1–18.6)	14.4 (11.6–21.6)	0.66
Skin color (n (%))				
Brown	27 (38.6)	12 (41.4)	15 (36.6)	0.59
White	22 (31.4)	10 (34.5)	12 (29.3)	
Black	16 (22.9)	5 (17.2)	11 (26.8)	
Yellow	1 (1.4)	1 (3.4)	0 (0)	
Unknown	4 (5.7)	1 (3.4)	3 (7.3)	
Marital status (n (%))				
Married	56 (82.3)	23 (79.3)	33 (84.6)	0.57
Single	12 (17.7)	6 (20.7)	6 (15.4)	
Education level (n (%))				
Up to 12 years schooling	46 (66.7)	20 (69.0)	26 (65.0)	0.73
Higher education	23 (33.3)	9 (31.0)	14 (35.0)	
Employment status (n (%))				
Employed	42 (60.9)	16 (55.2)	26 (65.0)	0.41
Unemployed	27 (39.1)	13 (44.8)	14 (35.0)	
Per capita income (US$ ^†^)	151.51 (103.04–227.27)	136.36 (91.67–221.04)	154.54 (113.33–228.78)	0.59
Housing Conditions (n (%))				
Adequate sanitation	64 (95.5)	27 (96.4)	37 (94.9)	1.00
Inadequate sanitation	3 (4.5)	1 (3.6)	2 (5.1)	
Parity	1 (0–1.25)	1 (0–1.5)	1 (0–1.5)	0.92
Pre-pregnancy BMI (kg/m^2^)	27.85 (24.4–32.3)	28.60 (25.7 –3.3)	27.10 (24.3–31.9)	0.16
Chronic hypertension (n (%))				
No	56 (84.8)	23 (79.3)	33 (89.2)	0.31
Yes	10 (15.2)	6 (20.7)	4 (10.8)	
Previous preeclampsia (n (%))				
No	63 (90.0)	25 (86.2)	38 (92.7)	0.38
Yes	7 (10.0)	4 (13.8)	7 (7.3)	
DM type (n (%))				
Type 1 DM	36 (51.4)	15 (51.7)	21 (51.2)	0.97
Type 2 DM	34 (48.6)	14 (48.3)	20 (48.8)	
Years since onset of DM	8 (2.0–13.5)	9 (2–14.5)	6 (1.9–12.5)	0.36
SBP (mmHg)	110 (100–120)	110 (100–120)	110 (100–120)	0.90
DBP (mmHg)	70 (62–80)	70 (70–80)	70 (60–80)	0.14
Calcium supplement (n (%))				
No	60 (85.7)	23 (79.3)	37 (90.2)	0.30
Yes	10 (14.3)	6 (20.7)	4 (9.8)	
HbA1c (%)	6.8 (6.2–7.9)	6.9 (6.4–8.0)	6.8 (6.1–7.7)	0.28
Development of HDP (n (%))				
GH	5 (7.14)	3 (10.3)	2 (4.9)	0.64
PE	11 (15.7)	4 (13.8)	7 (17.1)	0.75

DM: diabetes mellitus, BMI: body mass index, SBP: systolic blood pressure, DBP: diastolic blood pressure, HbA1c: serum glycated hemoglobin concentrations, HDP: hypertensive disorders of pregnancy, GH: gestational hypertension, PE: preeclampsia. † Estimated exchange rate of 1 real (R$) = 5.5 US dollars. Data presented as median (interquartile range) or as absolute and relative frequencies n (%). * Mann–Whitney U test or Kruskal–Wallis test to compare medians and Chi-square test to compare frequencies.

**Table 3:** FTO and ADRB2 polymorphisms in the study participants (Rio de Janeiro, Brazil, 2016–2020).

	Overall n = 70	DASH Diet n = 29	Traditional Diet n = 41	*p*-Value *
FTO rs9939609 (n (%))				
TT	28 (40.0)	11 (37.9)	17 (41.5)	0.48
AT	34 (48.6)	13 (44.8)	21 (51.2)	
AA	8 (11.4)	5 (17.2)	3 (7.3)	
FTO rs17817449 (n (%))				
TT	32 (45.7)	13 (44.8)	19 (46.3)	0.73
GT	31 (44.3)	12 (41.4)	19 (46.3)	
GG	7 (10.0)	4 (13.8)	3 (7.3)	
ADRB2 rs1042713 (n (%))				
GG	25 (35.7)	13 (44.8)	12 (29.3)	0.35
AG	37 (52.9)	14 (48.3)	23 (56.1)	
AA	8 (11.4)	2 (6.9)	6 (14.6)	
ADRB2 rs1042714 (n (%))				
CC	35 (50.0)	15 (51.7)	20 (48.3)	0.28
CG	31 (44.3)	14 (48.3)	17 (41.5)	
GG	4 (5.7)	0 (0)	4 (9.8)	

FTO: fat mass and obesity-associated gene, ADRB2: adrenoceptor beta 2 gene. Data presented as absolute and relative frequencies n (%). * Chi-square test or Fisher’s exact test to compare frequencies. Genotypes were in Hardy–Weinberg equilibrium.

**Table 4:** (**a**) Time-to-event analyses (from conception to development of HDP), analyzing dietetic and phenotypic characteristics. (**b**) Time-to-event analyses (from conception to development of HDP), analyzing blood pressure measurements and previous HDP.

Characteristics	Outcome	pY	Crude Incidence/ 100 pY (95% CI)	HR (95% CI)	*p* Value	aHR * (95% CI)	*p* Value
(a)							
Overall	16	49.22	32.51 (18.58–52.79)	-	-	-	-
Diet							
Traditional diet	9	28.38	31.71 (14.50–60.19)	Reference	-	Reference	-
DASH diet	7	20.83	33.60 (13.51–69.23)	0.98 (0.36–2.64)	0.97	0.81 (0.25–2.61)	0.72
Type of DM							
Type 1 DM	9	24.82	36.26 (16.58–68.84)	Reference	-	Reference	-
Type 2 DM	7	24.4	28.69 (11.54–59.12)	0.73 (0.27–1.96)	0.53	0.22 (0.04–1.12)	0.07
Age (years)							
<32	8	25.72	31.11 (13.43–61.30)	Reference	-	Reference	-
≥32	8	23.50	34.04 (14.7–67.08)	1.03 (0.39–2.76)	0.95	0.74 (0.23–2.35)	0.60
Color of skin							
Brown	5	19.40	25.78 (8.37–60.16)	Reference	-	Reference	-
White	3	15.17	19.77 (4.08–57.78)	0.73 (0.17–3.07)	0.67	1.10 (0.19–6.32)	0.91
Black	7	11.07	63.22 (25.42–130.26)	3.73 (1.17–11.91)	0.03	8.63 (1.85–40.18)	**0.01**
Pre-pregnancy BMI							
Normal weight	3	13.67	21.94 (4.52–64.12)	Reference	-	Reference	-
Overweight	6	17.57	34.14 (12.53–74.31)	1.80 (0.45–7.20)	0.41	2.69 (0.31–23.33)	0.37
Obesity	7	17.97	38.96 (15.66–80.27)	1.82 (0.47–7.03)	0.39	1.37 (0.18–10.49)	0.76
Gestational weight gain (kg)							
<12.2	9	24.28	37.07 (16.95–70.37)	Reference	-	Reference	-
≥12.2	7	24.24	28.88 (11.61–59.50)	0.82 (0.30–2.20)	0.69	1.47 (0.35–6.24)	0.60
Chronic diseases							
None	9	34.20	26.32 (12.03–49.96)	Reference	-	Reference	-
Chronic hypertension	3	6.36	47.15 (9.72–137.79)	1.73 (0.47–6.44)	0.41	1.93 (0.47–7.98)	0.36
Hypothyroidism	3	5.76	52.08 (10.74–152.2)	1.82 (0.49–6.73)	0.37	1.02 (0.13–7.93)	0.98
HbA1c 1st trimester (%)							
<7.77	3	10.69	28.05 (5.79–81.98)	Reference	-	Reference	-
≥7.77	3	10.78	27.84 (5.74–81.36)	0.89 (0.18–4.44)	0.89	1.28 (0.08–21.01)	0.86
HbA1c 2nd trimester (%)							
<6.47	4	22.49	17.78 (4.85–45.53)	Reference	-	Reference	-
≥6.47	9	21.71	41.45 (18.95–78.69)	3.33 (1.01–10.94)	0.05	2.26 (0.46–11.02)	0.31
HbA1c 3rd trimester (%)							
<6.41	3	19.55	15.35 (3.17–44.86)	Reference	-	Reference	-
≥6.41	6	18.24	32.89 (12.07–71.59)	2.75 (0.68–11.05)	0.15	4.76 (1.14–19.86)	**0.03**
(b)							
Overall	16	49.22	32.51 (18.58–52.79)	-	-	-	-
Previous HDP							
None	9	26.45	34.03 (15.56–64.59)	Reference	-	Reference	-
Preeclampsia	3	4.76	62.97 (12.99–184.04)	3.18 (0.85–11.95)	0.09	11.66 (2.22–61.07)	**<0.01**
SBP peak > 130 mmHg							
No	4	24.48	16.34 (4.45–41.84)	Reference	-	Reference	-
Yes	12	24.74	48.51 (25.07–84.74)	3.38 (1.09–10.48)	0.03	3.98 (0.96–16.41)	0.06
DBP peak > 80 mmHg							
No	0	11.62	0	Reference	-	Reference	-
Yes	16	37.60	42.55 (24.32–69.10)	-	-	-	-
MBP peak > 100 mmHg							
No	1	23.85	4.19 (0.11–23.36)	Reference	-	Reference	-
Yes	15	25.37	59.13 (33.1–97.53)	17.67 (2.33–134.04)	<0.01	18.42 (1.38–245.98)	**0.03**
SBP 1st trimester (mmHg)							
<111	4	11.45	34.94 (9.52–89.45)	Reference	-	Reference	-
≥111	6	12.02	49.92 (18.32–108.65)	1.55 (0.44–5.51)	0.50	1.70 (0.20–14.49)	0.63
SBP 2nd trimester (mmHg)							
<111	6	23.80	25.21 (9.25–54.87)	Reference	-	Reference	-
≥111	9	21.86	41.17 (18.83–78.16)	1.86 (0.66–5.24)	0.24	3.03 (0.71–12.86)	0.13
SBP 3rd trimester (mmHg)							
<114	3	25.31	11.85 (2.44–34.64)	Reference	-	Reference	-
≥114	13	23.91	54.38 (28.95–92.99)	5.81 (1.65–20.44)	0.01	5.56 (1.09–28.42)	**0.04**
DBP 1st trimester (mmHg)							
<70	3	12.90	23.25 (4.79–67.94)	Reference	-	Reference	-
≥70	7	10.57	66.25 (26.64–136.51)	3.31 (0.85–12.80)	0.08	70.15 (1.43–3450.19)	**0.03**
DBP 2nd trimester (mmHg)							
<69.7	7	23.79	29.42 (11.83–60.63)	Reference	-	Reference	-
≥69.7	8	21.87	36.58 (15.79–72.08)	1.40 (0.51–3.87)	0.51	1.54 (0.40–5.98)	0.53
DBP 3rd trimester (mmHg)							
<72	6	25.26	23.76 (8.72–51.71)	Reference	-	Reference	-
≥72	10	23.96	41.74 (20.01–76.76)	2.08 (0.75–5.73)	0.16	3.34 (0.81–13.76)	0.09

pY: person-years, CI: confidence interval, DASH: Dietary Approach to Stop Hypertension, HDP: hypertensive disorders of pregnancy, DM: diabetes mellitus, BMI: body mass index, HbA1c: serum glycated hemoglobin concentrations. Normal weight: BMI = 18.5–24.9 kg/m^2^, Overweight: BMI = 25–29.9 kg/m^2^, Obesity: BMI ≥ 30 kg/m^2^. Numerical variables categorized by the median of the sample. * Adjusted for skin color, previous HDP, household income, and years since onset of DM.

**Table 5:** Time-to-event analyses (from conception to development of HDP), analyzing genotypic characteristics: FTO and ADRB2 polymorphisms (rs9939609, rs17817449, rs1042713, and rs1042714).

	Outcome	pY	Crude Incidence/100 pY (95% CI)	HR (95% CI)	*p*-Value	aHR * (95% CI)	*p*-Value
Overall	16	49.22	32.51 (18.58–52.79)	-	-	-	-
FTO rs9939609							
TT	8	19.83	40.35 (17.42–79.5)	Reference	-	Reference	-
AT/AA	8	29.39	27.22 (11.75–53.64)	0.64 (0.24–1.71)	0.38	0.68 (0.22–2.10)	0.50
FTO rs17817449							
TT	10	22.67	44.11 (21.15–81.12)	Reference	-	Reference	-
GT/GG	6	26.55	22.60 (8.29–49.19)	0.46 (0.17–1.28)	0.14	0.63 (0.20–1.96)	0.42
ADRB2 rs1042713							
GG	4	17.80	22.47 (6.12–57.52)	Reference	-	Reference	-
AG/AA	12	31.41	38.20 (19.74–66.73)	1.60 (0.52–4.98)	0.41	1.59 (0.43–5.94)	0.49
ADRB2 rs1042714							
CC	8	24.40	32.79 (14.16–64.61)	Reference	-	Reference	-
GC/GG	8	24.82	32.23 (13.92–63.51)	1.05 (0.39–2.79)	0.93	1.87 (0.61–5.72)	0.27

Results for comparison between MAF carriers vs. MAF non-carriers. pY: person-years, CI: confidence interval, HDP: hypertensive disorders of pregnancy. MAF: minor allele frequency. FTO: fat mass and obesity-associated gene, ADRB2: adrenoceptor beta 2 gene. * Adjusted for DM type, color of skin, and previous HDP.

**Table 6:** Time-to-event analyses (from conception to development of HDP), analyzing the haplotypes ADRB2 rs1042713:rs1042714 and FTO rs9939609:rs17817449.

Characteristics	Outcome	pY	Crude Incidence/ 100 pY (95% CI)	HR (95% CI)	*p* Value	aHR * (95% CI)	*p* Value
ADRB2 rs1042713:rs1042714							
AC	13	36.45	35.67 (18.99–60.99)	-	-	-	-
GC	11	34.38	31.99 (15.97–57.24)	0.90 (0.40–2.01)	0.79	0.79 (0.34–1.82)	0.58
GG	8	27.60	28.98 (12.51–57.11)	0.88 (0.36–2.12)	0.77	1.09 (0.43–2.76)	0.85
FTO rs9939609:rs17817449							
TT	24	62.44	38.44 (24.63–57.19)	-	-	-	-
AG	6	30.91	19.41 (7.12–42.25)	0.46 (0.19–1.13)	0.08	0.52 (0.20–1.35)	0.24
AT	2	4.34	46.09 (5.58–166.48)	1.14 (0.27–4.84)	0.85	0.60 (0.11–3.35)	0.57

pY: person-years, CI: confidence interval, HDP: hypertensive disorders of pregnancy, FTO: fat mass and obesity-associated gene, ADRB2: adrenoceptor beta 2 gene. * Adjusted for DM type, color of skin, and previous HDP.

## Data Availability

Please, contact the correspondent author to verify the availability of the research data.

## Supplementary Materials

The following supporting information can be downloaded at: https://www.mdpi.com/article/10.3390/life13051104/s1. Table S1: Mean trajectory of blood pressure during pregnancy in the dietary groups. Table S2: Mean trajectory of blood pressure during pregnancy according to rs9939609 FTO genotype (A allele carriers vs. non-carriers) and interaction with diet. Table S3: Mean trajectory of blood pressure during pregnancy according to rs17817449 FTO genotype (G allele carriers vs. non-carriers) and interaction with diet. Table S4: Mean trajectory of blood pressure during pregnancy according to rs1042713 ADRB2 genotype (A allele carriers vs. non-carriers) and interaction with diet. Table S5: Mean trajectory of blood pressure during pregnancy according to rs1042714 ADRB2 genotype (G allele carriers vs. non-carriers) and interaction with diet.
