# Supplementary material for: A Pilot Study of Dietetic, Phenotypic, and Genotypic Features Influencing Hypertensive Disorders of Pregnancy in Women with Pregestational Diabetes Mellitus
Source: Life (Basel). 2023 Apr 28;13(5):1104. doi: 10.3390/life13051104 (PMC10220776; doi:10.3390/life13051104)
Supplement: Supplementary file 1 [file life-13-01104-s001.zip › life-2165602-supplementary.pdf]

Supplementary Table S1 – Mean trajectory of blood pressure during pregnancy in the diet groups.

		**< 6.5 weeks**	**6.5 to 19.5 weeks**	**19.5 to 32.5 weeks**	**> 32.5 weeks**	**p-value***
**SBP**	Trad. diet	121.09	115.30	117.03	131.08	0.71
		(113.89 – 128.28)	(108.89 – 121.71)	(110.86 – 123-20)	(122.43 – 139.72)	
	DASH diet	120.13	114.34	116.08	130.12	
		(113.24 – 127.03)	(108.26 – 120.43)	(110.24 – 121.91)	(121.72 – 138.53)	
**DBP**	Trad. diet	80.26	74.09	74.62	86.02	0.62
		(74.18 – 86.34)	(68.64 – 79.55)	(69.27 – 79.96)	(78.96 – 93.08)	
	DASH diet	79.42	73.25	73.78	85.18	
		(73.59 – 85.26)	(68.05 – 78.46)	(68.68 – 78.88)	(78.32 – 92.05)	

SBP: systolic blood pressure; DBP: diastolic blood pressure; Trad. diet: Traditional diet.
Data are expressed as mean (CI 95%).
* Adjusted for DM type, previous HDP, pre-pregnancy BMI, chronic disease (hypertension or hypothyroidism), and housing conditions.

Supplementary Table S2 – Mean trajectory of blood pressure during pregnancy according to rs9939609 FTO genotype (A allele carriers *vs*. non-carriers) and interaction with diet.

		< 6.5 weeks			6.5 to 19.5 weeks			19.5 to 32.5 weeks			> 32.5 weeks			p*[a]	p*[b]	p*[c]
		Overall	Trad Diet	DASH Diet	Overall	Trad Diet	DASH Diet	Overall	Trad Diet	DASH Diet	Overall	Trad Diet	DASH Diet			
**SBP**	TT	118.54 (111.36 – 125.72)	117.59 (109.46 – 125.73)	120.36 (111.69 – 129.03)	112.76 (106.37 – 119.16)	111.83 (104.40 – 119.26)	114.59 (106.55 – 122.63)	114.52 (108.36 – 120.67)	113.59 (106.37 – 120.82)	116.36 (108.48 – 124.24)	128.59 (119.93 – 137.25)	127.67 (118.21 – 137.13)	130.44 (120.48 – 140.41)	0.22	0.31	0.99
	AT/AA	121.52 (114.80 – 128.24)	123.06 (115.56 – 130.56)	119.40 (111.84 – 126.96)	115.74 (109.87 – 121.61)	117.29 (110.56 – 124.03)	113.63 (106.83 – 120.44)	117.50 (111.86 – 123.14)	119.06 (112.51 – 125.61)	115.40 (108.79 – 122.01)	131.57 (123.22 – 139.92)	133.14 (124.12 – 142.16)	129.48 (120.45 – 138.51)			
**DBP**	TT	77.67 (71.59 – 83.74)	76.58 (69.93 – 83.23)	78.84 (72.19 – 85.50)	71.47 (66.07 – 76.88)	70.38 (64.34 – 76.42)	72.65 (66.58 – 78.71)	71.99 (66.71 – 77.28)	70.91 (64.98 – 76.84)	73.17 (67.19 – 79.16)	83.44 (76.42 – 90.46)	82.39 (74.85 – 89.94)	84.66 (77.06 – 92.26)	0.06	0.08	0.99
	AT/AA	80.58 (74.91 – 86.25)	81.64 (75.60 – 87.67)	78.54 (72.44 – 84.64)	74.39 (69.43 – 79.34)	75.44 (70.08 – 80.80)	72.34 (66.89 – 77.80)	74.91 (70.07 – 79.75)	75.97 (70.70 – 81.24)	72.88 (67.51 – 78.24)	86.35 (79.62 – 93.08)	87.45 (80.36 – 94.54)	84.36 (77.23 – 91.48)			
**MBP**	TT	90.37 (83.79 – 96.96)	88.94 (81.69 – 96.18)	91.98 (84.73 – 99.24)	84.43 (78.33 – 90.54)	82.99 (76.20 – 89.79)	86.03 (79.21 – 92.87)	85.48 (79.51 – 91.46)	84.05 (77.36 – 90.74)	87.09 (80.35 – 93.84)	97.94 (90.23 – 105.66)	96.53 (88.22 – 104.84)	99.58 (91.21 – 107.94)	0.09	0.08	0.99
	AT/AA	93.37 (87.24 – 99.50)	94.61 (88.06 – 101.16)	90.93 (84.31 – 97.56)	87.43 (81.82 – 93.04)	88.67 (82.61 – 94.72)	84.99 (78.84 – 91.14)	88.48 (82.99 – 93.98)	89.73 (83.77 – 95.69)	86.05 (79.99 – 92.10)	100.94 (93.56 – 108.33)	102.21 (94.42 – 109.99)	98.53 (90.70 – 106.36)			

SBP: systolic blood pressure; DBP: diastolic blood pressure; MBP: mean blood pressure, FTO: fat mass and obesity-associated gene.
Data are expressed as mean (CI 95%).
* Adjusted for DM type, history of HDP, pre-pregnancy BMI, chronic disease (hypertension or hypothyroidism), and housing conditions.
a p value for comparison between the genotypes
b p value for comparison between genotypes in the Traditional Diet group
c p value for comparison between genotypes in the DASH Diet group

Supplementary Table S3 – Mean trajectory of blood pressure during pregnancy according to rs17817449 FTO genotype (G allele carriers *vs*. non-carriers) and interaction with diet.

		**< 6.5 weeks**			**6.5 to 19.5 weeks**			**19.5 to 32.5 weeks**			**> 32.5 weeks**			**p*a**	**p*b**	**p*c**
		Overall	Trad Diet	DASH Diet	Overall	Trad Diet	DASH Diet	Overall	Trad Diet	DASH Diet	Overall	Trad Diet	DASH Diet			
**SBP**	TT	119.55 (112.39-126.70)	119.40 (111.09-127.71)	119.76 (111.54-127.99)	113.77 (107.40-120.14)	113.62 (105.99-121.25)	113.98 (106.44-121.52)	115.52 (109.40-121.64)	115.38 (107.95-122.80)	115.74 (108.40-123.08)	129.58 (120.98-138.18)	129.46 (119.89-139.03)	129.82 (120.30-139.35)	0.52	0.84	0.99
	GT/GG	121.08 (114.30-127.87)	122.05 (114.43-129.67)	119.86 (111.97-127.74)	115.30 (109.35-121.26)	116.27 (109.39-123.14)	114.08 (106.89-121.26)	117.05 (111.33-122.78)	118.02 (111.35-124.70)	115.83 (108.84-122.83)	131.12 (122.73-139.50)	132.11 (123.03-141.19)	129.92 (120.64-139.20)			
**DBP**	TT	78.61 (72.69-84.53)	78.88 (71.96-85.80)	78.27 (72.00-84.53)	72.43 (67.16-77.69)	72.68 (66.31-79.06)	72.07 (66.42-77.72)	72.95 (67.80-78.11)	73.21 (66.93-79.50)	72.60 (67.05-78-15)	84.41 (77.49-91.32)	84.69 (76.89-92.49)	84.07 (76.85-91.30)	0.19	0.67	0.92
	GT/GG	80.65 (74.83-86.47)	81.26 (74.99-87.54)	79.99 (73.26-86.71)	74.47 (69.32-79.62)	75.07 (69.42-80.71)	73.79 (67.60-79.98)	75.00 (69.94-80.06)	75.60 (70.04-81.16)	74.32 (68.19-80.45)	86.45 (79.56-93.34)	87.07 (79.79-94.36)	85.79 (78.11-93.48)			
**MBP**	TT	91.44 (85.03-97.86)	91.25 (83.66-98.84)	91.28 (84.47-98.10)	85.50 (79.56-91.44)	85.30 (78.11-92.49)	85.33 (78.96-91.69)	86.56 (80.75-92.37)	86.36 (79.26-93.45)	86.39 (80.13-92.64)	99.02 (91.44-106.60)	98.84 (90.24-107.45)	98.87 (90.93-106.81)	0.27	0.64	0.99
	GT/GG	93.41 (87.09-99.72)	94.05 (87.20-100.90)	92.22 (84.83-99.60)	87.47 (81.63-93.30)	88.10 (81.70-94.49)	86.27 (79.27-93.26)	88.52 (82.79-94.25)	89.15 (82.85-95.45)	87.32 (80.41-94.24)	100.99 (93.42-108.55)	101.64 (93.62-109.66)	99.81 (91.32-108.30)			

SBP: systolic blood pressure; DBP: diastolic blood pressure; MBP: mean blood pressure, FTO: fat mass and obesity-associated gene.
Data are expressed as mean (CI 95%).
* Adjusted for DM type, history of HDP, pre-pregnancy BMI, chronic disease (hypertension or hypothyroidism), and housing conditions.
a p value for comparison between the genotypes
b p value for comparison between genotypes in the Traditional Diet group
c p value for comparison between genotypes in the DASH Diet group

Supplementary Table S4 – Mean trajectory of blood pressure during pregnancy according to rs1042713 ADRB2 genotype (A allele carriers *vs*. non-carriers) and interaction with diet.

		< 6.5 weeks			6.5 to 19.5 weeks			19.5 to 32.5 weeks			> 32.5 weeks			p*a	p*b	p*c
		Overall	Trad Diet	DASH Diet	Overall	Trad Diet	DASH Diet	Overall	Trad Diet	DASH Diet	Overall	Trad Diet	DASH Diet			
**SBP**	GG	119.36 (111.91-126.80)	121.57 (112.65-130.49)	117.21 (108.81-125.60)	113.56 (106.84-120.28)	115.81 (107.45-124.16)	111.45 (103.67-119.22)	115.31 (108.84-121.79)	117.59 (109.44-125.74)	113.23 (105.69-120.76)	129.40 (120.60-138.20)	131.71 (121.64-141.79)	127.35 (117.77-136.93)	0.50	0.99	0.58
	AG/ AA	121.02 (114.31-127.73)	120.57 (113.10-128.04)	122.19 (114.57-129.80)	115.22 (109.34-121.11)	114.81 (108.07-121.55)	116.43 (109.49-123.36)	116.97 (111.36-122.59)	116.59 (110.11-123.07)	118.21 (111.50-124.92)	131.06 (122.80-139.32)	130.71 (121.87-139.56)	132.33 (123.32-141.34)			
**DBP**	GG	80.19 (74.15-86.23)	80.82 (73.99-87.66)	79.93 (73.21-86.65)	74.04 (68.60-79.47)	74.66 (68.35-80.97)	73.77 (67.61-79.93)	74.56 (69.23-79.88)	75.18 (68.96-81.40)	74.29 (68.24-80.34)	85.93 (78.93-92.94)	86.57 (78.87-94.28)	85.68 (78.11-93.25)	0.68	0.99	0.99
	AG/ AA	79.52 (73.66-85.37)	80.07 (73.67-86.46)	79.09 (72.83-85.35)	73.36 (68.16-78.57)	73.91 (68.13-79.68)	72.93 (67.25-78.61)	73.88 (68.79-78.97)	74.43 (68.76-80.09)	73.45 (67.85-79.04)	85.26 (78.39-92.13)	85.82 (78.51-93.13)	84.84 (77.60-92.08)			
**MBP**	GG	92.43 (85.87-98.99)	93.66 (86.14-101.19)	91.53 (84.18-98.87)	86.50 (80.37-92.62)	87.75 (80.60-94.90)	85.61 (76.68-92.55)	87.54 (81.54-93.54)	88.82 (81.77-95.86)	86.68 (79.87-93.49)	99.97 (92.29-107.66)	101.26 (92.73-109.78)	99.12 (90.78-107.45)	0.94	0.99	0.98
	AG/ AA	92.57 (86.23-98.91)	92.77 (85.80-99.75)	92.66 (85.82-99.50)	86.64 (80.77-92.51)	86.86 (80.34-93.39)	86.75 (80.32-93.17)	87.68 (81.94-93.43)	87.93 (81.53-94.33)	87.81 (81.49-94.12)	100.11 (92.58-107.65)	100.37 (92.31-108.42)	100.25 (92.28-108.22)			

SBP: systolic blood pressure; DBP: diastolic blood pressure; MBP: mean blood pressure, ADRB2: adrenoceptor beta 2 gene.
Data are expressed as mean (CI 95%).
* Adjusted for DM type, history of HDP, pre-pregnancy BMI, chronic disease (hypertension or hypothyroidism), and housing conditions.
a p value for comparison between the genotypes
b p value for comparison between genotypes in the Traditional Diet group
c p value for comparison between genotypes in the DASH Diet group

Supplementary Table S5 – Mean trajectory of blood pressure during pregnancy according to rs1042714 ADRB2 genotype (G allele carriers *vs*. non-carriers) and interaction with diet.

		< 6.5 weeks			6.5 to 19.5 weeks			19.5 to 32.5 weeks			> 32.5 weeks			p^{*a}	p^{*b}	p^{*c}
		Overall	Trad Diet	DASH Diet	Overall	Trad Diet	DASH Diet	Overall	Trad Diet	DASH Diet	Overall	Trad Diet	DASH Diet			
**SBP**	CC	119.34 (112.55-126.12)	120.93 (112.93-128.92)	118.29 (110.77-125.81)	113.53 (107.54-119.53)	115.14 (107.81-122.47)	112.51 (105.71-119.30)	115.24 (109.48-121.01)	116.89 (109.75-124.03)	114.25 (107.70-120.81)	129.24 (120.87-137.61)	130.95 (121.56-140.34)	128.32 (119.41-137.22)	0.27	0.98	0.61
	GC/ GG	121.94 (114.96-128.92)	122.02 (114.18-129.86)	122.90 (114.60-131.19)	116.14 (109.93-122.35)	116.23 (109.10-123.37)	117.11 (109.44-124.78)	117.85 (111.89-123.81)	117.98 (111.09-124,87)	118.86 (111.38-126.34)	131.85 (123.38-140.31)	132.04 (122.92-141.17)	132.92 (123.31-142.53)			
**DBP**	CC	78.96 (73.24-84.67)	80.19 (73.69-86.68)	78.32 (72.31-84.32)	72.78 (67.72-77.84)	74.01 (68.09-79.92)	72.14 (66.75-77.52)	73.27 (68.31-78.23)	74.52 (68.68-80.35)	72.65 (67.37-77.93)	84.61 (77.82-91.41)	85.91 (78.42-93.40)	84.04 (77.02-91.06)	0.15	0.89	0.49
	GC/ GG	81.22 (75.25-87.20)	81.82 (75.18-88.06)	81.79 (75.05-88.53)	75.04 (69.70-80.39)	75.44 (69.61-81.27)	75.61 (69.40-81.81)	75.53 (70.30-80.77)	75.95 (70.23-81.66)	76.12 (69.99-82.25)	86.88 (79.91-93.85)	87.34 (80.00-94.69)	87.51 (79.82-95.20)			
**MBP**	CC	91.66 (85.49-97.83)	93.17 (86.05-100.29)	91.01 (84.49-97.53)	85.71 (80.01-91.41)	87.23 (80.53-93.93)	85.07 (79.00-91.13)	86.73 (81.14-92.31)	88.27 (81.66-94.88)	86.11 (80.17-92.05)	99.10 (91.68-106.52)	100.70 (92.45-108.95)	98.54 (90.86-106.22)	0.17	0.93	0.50
	GC/ GG	94.07 (87.58-100.56)	94.52 (87.47-101.57)	94.91 (87.48-102.34)	88.13 (82.09-94.16)	88.58 (81.97-95.19)	88.97 (81.93-96.02)	89.14 (83.22-95.06)	89.62 (83.14-96.10)	90.01 (83.06-96.97)	101.51 (93.87-109.16)	102.05 (93.96-110.15)	102.44 (93.94-110.94)			

SBP: systolic blood pressure; DBP: diastolic blood pressure; MBP: mean blood pressure, ADRB2: adrenoceptor beta 2 gene.
Data are expressed as mean (CI 95%).
* Adjusted for DM type, history of HDP, pre-pregnancy BMI, chronic disease (hypertension or hypothyroidism), and housing conditions.
a p value for comparison between the genotypes
b p value for comparison between genotypes in the Traditional Diet group
c p value for comparison between genotypes in the DASH Diet group
